# Quantification of Minerals in Edible Mushrooms via Optimized Microwave-Assisted Digestion: Nutritional Contributions of Fe, Mg, Na, K, and Ca

**DOI:** 10.3390/foods13244051

**Published:** 2024-12-15

**Authors:** Alejandro R. López, Elena Ortega-Caneda, Estrella Espada-Bellido, Oscar R. Taracena-Zepeda, Miguel Palma, Gerardo Fernández-Barbero

**Affiliations:** Department of Analytical Chemistry, Faculty of Sciences, Agrifood Campus of International Excellence (ceiA3), Instituto de Investigación Vitivinícola y Agroalimentaria (IVAGRO), University of Cadiz, Puerto Real, 11510 Cadiz, Spain; alejandro.ruizlopez@alum.uca.es (A.R.L.); elena.ortegacaneda@alum.uca.es (E.O.-C.); miguel.palma@uca.es (M.P.); gerardo.fernandez@uca.es (G.F.-B.)

**Keywords:** mushrooms, minerals, microwave-assisted digestion, food supplements, Box–Behnken design, recommended daily intake

## Abstract

The intake of mushrooms provides numerous beneficial properties for the correct functioning of the human body due to their rich content in carbohydrates, proteins, fibers, vitamins, and minerals. However, most of the reports are focused on the determination of bioactive compounds and only a few regarding the essential mineral content and the evaluation of the RDI. Thus, the aim of this study was to determine the mineral composition of different cultivated (*A. bisporus* and *P. ostreatus*) and wild edible mushrooms (*A. crocodilinus*, *A. arvensis*, *A. silvicola*, *A. impudicus*, *M. mastoidea*, *M. rhacodes,* and *P. ostreatus*) collected in the south of Spain and north of Morocco. First, the optimization of a microwave-assisted digestion method was carried out using a Box–Behnken design with a response surface methodology to quantify the total content of five metals: Fe, Mg, Na, K, and Ca in mushrooms. The samples were analyzed by FAAS and ICP-OES. The percentage of the RDI of each mineral covered by the intake of mushrooms was calculated. It was observed that a high percentage of RDI levels are covered and just exceeded for Fe. Thence, due to their beneficial properties and high content of essential minerals, mushrooms would be proposed as a valuable source of nutrients to manufacture some food supplements.

## 1. Introduction

In today’s society, people are increasingly mindful of the potential side effects of medications and are seeking innovative natural compounds as alternative therapies. That fact, coupled with the idea of a balanced and enriched diet to improve life expectancy, is leading to the introduction of functional foods and even food supplements in the diet. Among the wide variety of foods, it has been known for centuries that some edible mushrooms have great features, like anti-inflammatory, liver protection, antioxidants, cholesterol-lowering properties, anti-diabetic and anti-microbial potential, or anti-carcinogenic properties due to their high content of both bioactive compounds and essential metals. For this reason, recently, the population is incorporating new functional foods, such as mushrooms, that provide several beneficial properties to ensure the correct functioning of the human body and could have a great potential as food supplements [1,2,3,4,5].

Although there are more than 110,000 species of macroscopic fungi in nature, only 78 of them are marketable as food in Spain, and only some are cultivated for subsequent commercialization, such as mushrooms of the genus *Agaricus* or *Pleurotus* [6]. Nevertheless, at the national level, the demand for wild mushrooms, including *Macrolepiota procera* and species from the *Lactarius* or *Agaricus* genera, has grown significantly in recent years, according to the Spanish Agency of Food Health and Nutrition, with an average consumption of 5.61 g per capita per day [7,8]. This increase has meant that its commercial value is reaching, and may even exceed, the commercial value of wood [9]. These mushrooms of the genus *Agaricus*, *Pleurotus*, and *Macrolepiota* are some of the most typically incorporated into the diet due to their annual availability on the market and their excellent culinary properties [10]. It is therefore of particular interest to study the nutritional mineral composition of these mushrooms to know the beneficial properties for health derived from their consumption and their potential use for the manufacture of food supplements.

Among all the edible mushrooms available to the consumer, cultivated and wild-growing mushrooms are characterized by accumulating a high level of minerals that are found in the fruiting soil [6]. Some of these minerals, like iron (Fe), magnesium (Mg), potassium (K), calcium (Ca), or sodium (Na), are crucial for the proper functioning of the human body, as they play a role in various functions and biological processes within the organism [11,12,13].

K is the primary intracellular cation in the human body, and it takes part in numerous functions, including nerve transmission and regulation of vascular tone. Maintaining proper K levels is crucial to prevent issues such as muscle weakness, cardiac arrhythmias, kidney stones, and an increased risk of heart attacks [14]. In addition, Mg is involved in many enzymatic processes. In fact, a Mg deficiency leads to specific biochemical abnormalities such as latent tetany, seizures, and numerous chronic diseases [15,16]. It is well-known that Fe is the most important trace element to ensure the proper functioning of the organism. For this reason, it is crucial to avoid depletion of this element [11]. On the other hand, Ca is the most abundant mineral in the human body, due to it being the main component of the bones and teeth; thus, low concentrations of this metal can lead to osteoporosis [17]. Finally, Na is the primary extracellular cation and also plays an important role in the membrane potential of cells. Overall, there is a little evidence of any adverse effects of low dietary sodium intake; however, an excess of sodium in the body can elevate the blood pressure, which is related to several cardiovascular diseases [11].

As it has been mentioned above, these minerals must be incorporated into the human body through diet. Thus, many authors have analyzed different food samples in order to quantify their mineral content, highlighting the articles carried out by Cabrera et al., where Fe has been determined in different types of legumes and nuts [18], or this reported by Vélez-Terreros et al., focused on the determination of different elements of nutritional interest in tomatoes [19]. Edible mushrooms are a great source of essential minerals of vital importance to human health; however, most articles referring to mushrooms are focused on the quantification of bioactive compounds. For instance, S. Reis. et al., consider mushrooms as a functional food due to their immunomodulatory antioxidant properties derived from the content of polyphenols, carotenoids, or polysaccharides [20]. As well as Heleno et al., who focus on the nutritional value of mushrooms on the content of fatty acids, tocopherols, and organic acids [21]. However, many authors also highlight the abundant mineral content present in mushrooms, which contribute to meeting the levels of essential metals that must be ingested through the diet [22,23,24]. As far as we know, there are only a few reports focused on the essential mineral composition and the evaluation of the RDI.

For that reason, it is important to quantify the total content of essential metals in mushrooms [25]. To carry out an optimum determination of these metals, the digestion of the organic matrix plays a fundamental role in the analytical process. The digestion of the samples must be very efficient, since an excess of organic matter in the digests can interfere with the quality of the results. To align with eco-friendly approaches, current methods prioritize the use of less acid, time, energy, and cost. Traditional dry digestion, which involves high temperatures and analyte loss, is being replaced by microwave-assisted wet digestion in closed vessels [26,27,28,29]. This approach minimizes volatilization, reduces residues, lowers energy consumption, speeds up dissolution, and enhances organic matter elimination.

It is important to highlight that the development of efficient and precise treatment samples and determination methods for nutrients in food matrices is crucial. The choice of the analytical methodology used to carry out the digestion of samples will depend on several factors, such as the nature of the matrix and the analyte, the available resources, the environmental commitment, and the analytical technique used to determine the analyte of interest. Most authors use digestion assisted by microwaves to determine the mineral content in mushrooms. However, they do not normally optimize the digestion methods in order to apply milder conditions [30,31,32,33,34].

Therefore, the aims of this research are: (1) the optimization of a microwave-assisted digestion method for mushrooms; (2) the determination of five essential minerals to the human body (Fe, Mg, K, Ca, and Na) present in highly consumed edible wild and cultivated mushroom samples; (3) the evaluation of the percentage of the recommended daily intake (RDI) of each element that is covered by the consumption of these mushrooms. Thus, this report pretends to bring to light information about the nutritional profile of mushrooms and their beneficial properties to the organism and to highlight their high potential value for the development of food supplements.

## 2. Materials and Methods

### 2.1. Mushroom Samples

**Figure 1:**
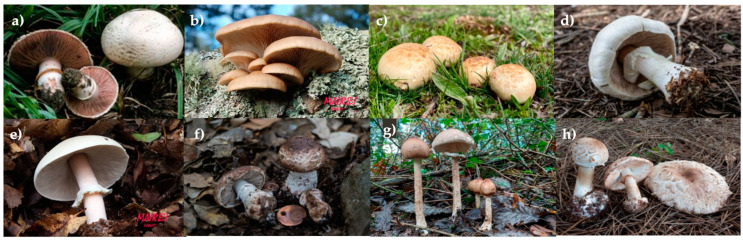
Cultivated and wild species of mushrooms: (**a**) *A. bisporus*; (**b**) *P. ostreatus*; (**c**) *A. crocodilinus*; (**d**) *A. arvensis*; (**e**) *A. silvicola* (**f**) *A. impudicus*; (**g**) *M. mastoidea*; (**h**) *M. rhacodes*. Pictures: Asociación Micológica del Estrecho (Mairei).

To carry out this study, a total of twelve samples of different genus and species of cultivated and wild edible mushrooms were analyzed (Table 1). Five of them were cultivated mushrooms (codified as #C1–#C5) comprising the species: *Agaricus bisporus* and *Pleurotus ostreatus*. On the other hand, the species of wild edible mushrooms (codified as #W6–#W12) were: *Agaricus crocodilinus*, *Agaricus arvensis*, *Agaricus silvicola*, *Agaricus impudicus*, *Macrolepiota mastoidea*, *Macrolepiota rhacodes,* and *Pleurotus ostreatus* (Figure 1). The specimens were recognized by mycologist experts using their distinctive morphological characteristics specific to each species.

The cultivated mushrooms were obtained from four different supermarket franchises commonly found in Spain and from Micotime (Cortes de la Frontera, Spain), a Spanish consulting company that gives services related to the knowledge, identification, conservation, and sustainable use of mycological resources. Wild samples were collected from various locations in southern Spain, specifically the provinces of Malaga and Granada, as well as from areas in northern Morocco, near the city of Tangier. To ensure a representative sample, at least 10 specimens were collected from each area. Before analysis, the samples underwent proper pretreatment, which included washing with deionized water, freeze-drying, and grinding into a fine powder using an agate pestle and mortar. The freeze-dried samples were then stored in well-labeled polyethylene (PE) bottles, indicating the species and sampling location.

### 2.2. Chemicals, Reagents and Reference Materials

To carry out the acid digestions, the reagents were purchased from SCP Science (Montreal, QC, Canada): HNO_3_ PlasmaPURE (67–69%), and from Sigma-Aldrich (St. Louis, MO, USA): H_2_O_2_ (≥30%). To prepare the multielement reference analytical solutions, high purity stock solutions of 1000 mg L^−1^ from SCP Science (Montreal, QC, Canada) of each metal were used. On the other hand, it was necessary the use of a buffer to eliminate interferences related to the magnesium measurement, which was purchased from Merck KGaA (Darmstadt, Germany): CsCl, LaCl_3_ (100%). Furthermore, all the solutions were prepared using ultrapure water obtained by passing twice-distilled water through a Milli-Q system (18 MΩ/cm, Millipore, Bedford, MA, USA). In addition, to verify the accuracy of the analytical procedure for all the determined elements, a certified reference material (CRM), specifically the CRM “*Tea leaves INCTL-TL-1*” purchased from the Institute of Nuclear Technology and Chemistry (Warsaw, Poland), was used. Finally, in order to correct temporal variations that could affect the signal intensity, an internal standard of ^45^Sc and ^72^Ge was used. That standard was prepared using stock individuals’ solutions (SCP Science; Montreal, QC, Canada) of 1000 μg mL^−1^.

### 2.3. Box–Behnken Design to Optimize the Digestion Method

The acid digestions were carried out using a microwave closed-vessel system acquired from Milestone SRL (Sorisole, Bergamo, Italy). That instrument is equipped with 15 positions for Teflon vessels with temperatures up to 300 °C and pressure of 100 bars. Furthermore, the equipment includes a touch panel that allow one to establish temperature ramps and digestion times to carry out the analysis. To obtain the optimal conditions for mushroom digestion, a Box–Behnken design (BBD) was carried out. This design uses statistical tools centered around a central point to develop a quadratic fitting model. The Box–Behnken design predicts the values of the dependent variables with fewer experiments, avoiding extreme conditions. This is achieved by eliminating axial points, resulting in a more spherical arrangement of the design points. In this case, a Box–Behnken design of three independent variables (volume of nitric acid, time, and temperature), five response variables (concentration of Fe, Mg, K, Ca, and Na) of one block, and fifteen runs (including three center points) was established using a pool of mushrooms belonging to the same species that have been analyzed in this study. The independent variables were normalized and coded at three levels as −1, 0, and +1. The ranges of the independent variables are shown below (Table 2).

The acid digestion procedure here employed was optimized for the volume of HNO_3_, the temperature and the time of digestion by taking the different values shown in Table 3. Samples were accurately weighed approximately 0.5 g directly in the microwave vessels. They were subjected to fifteen different digestion procedures using different conditions but keeping the same solid sample—reagent volume ratio (0.5 g to 10 mL). In addition to the volume of HNO_3_ used in each experiment, all the digestions were carried out by employing 1 mL of H_2_O_2_.

Once all the experiments were carried out, the data obtained were subjected to a statistical analysis to determine the optimal digestion conditions. Both individual optimization for each metal and a multiple response optimization were carried out. To develop the individual optimizations, firstly, an analysis of the variance (ANOVA) was conducted to assess the impact of the variables and the interactions among them. After that, a response surface method was used, from which a mathematical model is obtained, governed by a second-order equation, as shown in Equation (1).

(1) $$y=\beta_{0}+\sum_{i=1}^{k}\beta_{i}X_{i}+\sum_{i=1}^{k}\beta_{ii}X_{i}^{2}+\sum_{i=1}^{k}\sum_{j=1}^{k}\beta_{ij}X_{i}X_{j}+\varepsilon$$

This equation represents the system’s response as a function of the independent variables and their interactions. Here, *y* is the response variable (concentration of each metal), *β_i_* is the coefficient for each main effect, *β_ii_* is the coefficient for the quadratic factors that represent the curvature of the surface, *β_ij_* is the coefficient of the interactions between *i* and *j*, *X* denotes the studied factors, and *ε* is the residual value or random error.

The multiple response optimization was also carried out to obtain the optimal digestion conditions for the five metals, using the desirability approach. Each estimated response was transformed into a dimensionless scale ranging between 0 ≤ *d_i_* ≤ 1, where *d_i_* means desirability.

To obtain the overall desirability *D* (Equation (2)), the geometric average of each individual desirability value was combined. An algorithm was then applied to the *D* function to identify the set of variable values that maximize it [35].

*D* = (*d*_1_·*d*_2_·…·*d*_m_)^1/*m*^ (2)

where *m* means the number of responses. The *t*-test was used to compare the results obtained from the independent and the multi-response methods.

### 2.4. Digestion Procedure of Mushroom Samples

The optimal digestion procedure was carried out as follows: 0.5 g of freeze-dried sample was weighed, and then 5 mL of HNO_3_ and 1 mL of H_2_O_2_ were added, followed by nanopure water to a final volume of 10 mL. The samples were digested using a stepwise temperature increase over 20 min, reaching 198 °C, and this temperature was maintained for an additional 10 min. After digestion, the samples were filtered through a 0.45 μm filter with a −600 mbar vacuum port and transferred to a 50 mL volumetric DigiTube, filled to 50 mL with nanopure water. All samples were prepared in duplicate.

### 2.5. Instrumentation

The contents of Mg and Fe contained in the samples of mushrooms were determined using a flame atomic absorption spectrometer (FAAS) (Ice 3000 Series AA; Thermo Fisher Scientific; Walthman, MA, USA). This instrument featured a double-bundle optical system, with all lenses covered in silica and sealed to prevent the entry of ash. It also included an automatic alignment carousel capable of holding 6 hollow cathode lamps and a 50 mm universal titanium burner. The instrumental parameters, including burner height, fuel flow, slip width, and wavelength, were optimized for determining the content of each element, as detailed in Table 4.

On the other hand, the determination of Na, K, and Ca was performed using an Inductively Coupled Plasma Optical Emission Spectrometer (ICP-OES) (Spectrogreen FMX46; SPECTRO Analytical Instruments; Kleve, Dusseldorf, Germany). The ICP-OES was an automatic optical emission spectrometer that enabled simultaneous measurements by utilizing inductively coupled plasma excitation (argon ions) and a semiconductor-based detector system for liquid analysis. It provides a broad spectral analysis range from 165 to 770 nm.

### 2.6. Recommended Daily Intake of Metals

Macronutrients are essential for the proper functioning of the body, and there are some specific values of these metals that humans need, known as the recommended daily intake (RDI). Deviations from the RDI, either above or below, can result in various health issues and increase the risk of numerous diseases. To assess whether the consumption of the studied mushrooms, both wild and cultivated, satisfies the recommended daily intake, the mineral content determined in the mushrooms was compared with the RDI values for each element: K, Mg, Ca, Fe, and Na.

The Estimated Daily Intake (EDI) of each element provided by the studied mushrooms was calculated using the following formula:

EDI = *M* × *A* (3)

where *M* is the metal concentration in the mushroom samples, expressed in mg Kg^−1^, and *A* is the amount of dry matter consumed from the mushrooms, measured in Kg of dry matter per day. It was assumed that a tolerable daily intake of 300 g of fresh mushrooms, containing 30 g of dry matter, is consumed [36].

The contribution of all mushroom samples analyzed to the recommended daily intake was calculated as the percentage of the EDI of each metal compared to the RDI value according to the Spanish Federation of Nutrition, Food and Dietetic Societies (FESNAD) [37], using the following equation:

C = (EDI/RDI) × 100(4)

where EDI is the estimated daily intake of each element provided by the mushrooms, expressed in mg day^−1^, and RDI is the recommended daily intake, expressed in mg day^−1^.

### 2.7. Data Analysis and Software

To obtain the data from the instrumental equipment, the following software were used: ICP Analyzer-Pro version 1.40.0066e (Kleve, Dusseldorf, Germany) for ICP-OES and Solaar version 11.10 (Waltham, MA, USA) for FAAS. For the univariate statistical analysis, STATGRAPHICS Centurion 18 version 18.1.16 (StatPoint Technologies Inc., Warrenton, VA, USA) was used. All the other graphs presented in this work were created using Microsoft Office software (Redmond, WA, USA).

## 3. Results and Discussion

### 3.1. Digestion Optimization and Box–Behnken Design

Once all the experiments belonging to the Box–Behnken design were concluded, a statistical analysis of the data was carried out. The analysis of the variance (ANOVA) was used to obtain information about the mathematical model generated from the experimental data. As a result of this analysis, the full second-order equations obtained for each metal (Equations (5)–(9)) display the response as a function of the independent variables and their interactions:
**Fe Concentration** = 481.68 − 63.00 × HNO_3_ Volume − 45.29 × Time + 114.84 × Tem perature + 4.93 × HNO_3_ Volume^2^ + 5.39 × HNO_3_ Volume × Time − 135.45 × HNO_3_ Volume × Temperature + 8.73 × Time^2^ − 101.73 × Time × Temperature + 57.85 × Temperature^2^
(5)**Mg Concentration** = 973.57 − 112.74 × HNO_3_ Volume − 107.65 × Time + 300.09 × Temperature + 22.04 × HNO_3_ Volume^2^ − 8.84 × HNO_3_ Volume × Time − 222.18 × HNO_3_ Volume × Temperature + 12.62 × Time^2^ − 232.57 × Time × Temperature + 186.37 × Temperature^2^
(6)**Na Concentration** = 72.21 − 18.57 × HNO_3_ Volume − 7.70 × Time + 35.23 × Tem perature + 9.35 × HNO_3_ Volume^2^ − 2.67 × HNO_3_ Volume × Time − 30.60 × HNO_3_
Volume × Temperature − 3.16 × Time^2^ − 19.20 × Time × Temperature + 26.74 × Temperature^2^
(7)**K Concentration** = 23,250.50 − 3610.14 × HNO_3_ Volume − 3161.98 × Time + 6562.61 × Temperature − 30.81 × HNO_3_ Volume^2^ + 377.55 × HNO_3_ Volume × Time − 6591.32 × HNO_3_ Volume × Temperature + 337.91 × Time^2^ − 6249.70 × Time × Temperature + 6033.74 × Temperature^2^
(8)**Ca Concentration** = 478.59 − 104.79 × HNO_3_ Volume − 62.38 × Time + 178.78 × Temperature + 54.24 × HNO_3_ Volume^2^ + 2.00 × HNO_3_ Volume × Time − 183.88 × HNO_3_ Volume × Temperature − 21.12 × Time^2^ − 133.29 × Time × Temperature + 162.57 × Temperature^2^
(9)


**Figure 2:**
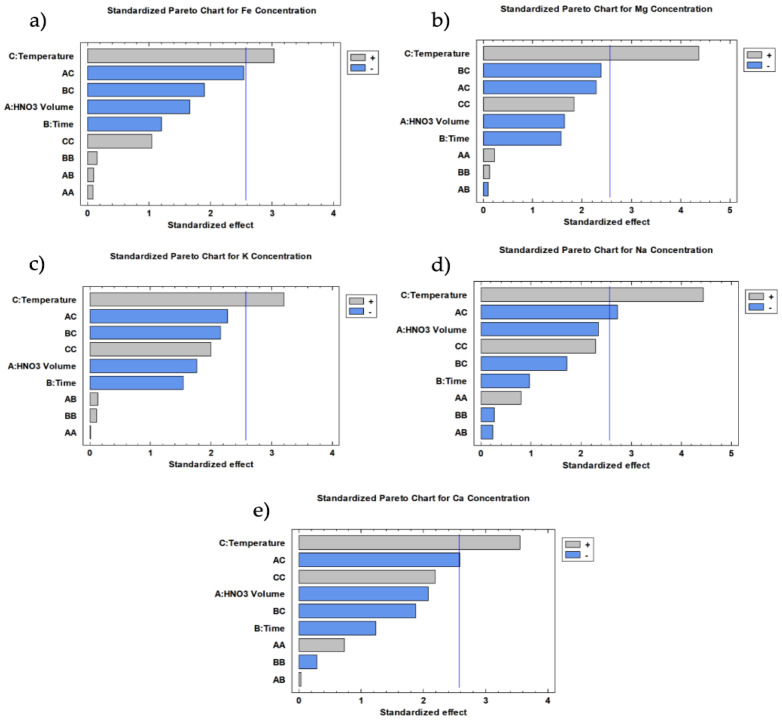
Standardized Pareto’s Chart for each element studied: (**a**) Fe; (**b**) Mg; (**c**) K; (**d**) Na; (**e**) Ca.

It is possible to reduce the equations using only the main effects that contribute statistically to the model. Thus, in the case of iron, magnesium, and potassium, temperature is the only variable that contributed statistically due to showing a *p*-value less than 0.05 at a confidence level of 95%. As far as calcium and sodium are concerned, both temperature and its interaction with the HNO_3_ volume were the variables that contributed statistically. For a better understanding, these contributions have been graphically described using the Pareto Charts of each metal, which are shown in Figure 2. These results are in accordance with the literature on other different food samples, such as beans, rice, and powdered milk, where temperature, acid volume, and the interaction between them are the variables that significantly affect the digestion [34,38,39].

**Figure 3:**
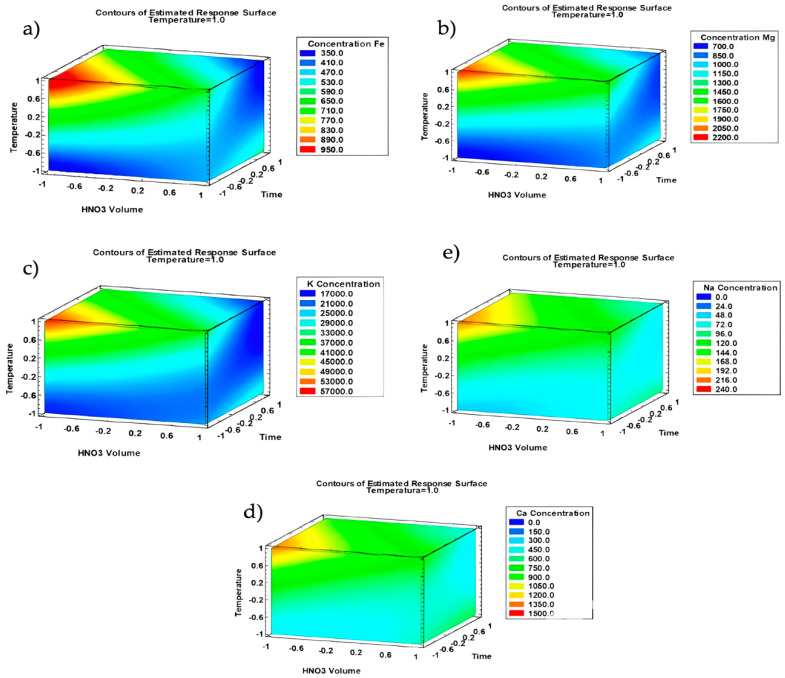
Three-dimensional surface graph of the Box–Behnken design representing the effects of the different interactions on the metal concentration: (**a**) Fe; (**b**) Mg; (**c**) K; (**d**) Ca; (**e**) Na.

Due to the clear effect of the temperature, the volume of nitric acid and the digestion time were evaluated fixing the temperature at its maximum value (200 °C), obtaining the corresponding response surface plots for each metal, which are shown in Figure 3.

The multiple response optimization was also carried out to obtain the compromise optimal digestion conditions for the five essential metals using the desirability approach. After performing the statistical analyses, it is concluded that both the individual and the multiple optimum conditions coincide, being the optimum value for temperature 198 °C, 10 min for time, and 5 mL for volume of nitric acid.

### 3.2. Method Validation

According to the International Organization for Standardization (ISO), the International Council for Harmonization (ICH) [40], and Eurachem guidelines [41], five different analytical parameters were calculated to validate the new method: limit of detection, limit of quantification, trueness, repeatability, and intermediate precision.

Firstly, the limit of detection (LOD) and quantification (LOQ) were calculated based on measurements of 10 sample blanks, using the following equations:

LOD = x_m_ + 3·SD (10)

LOQ = 3·LOD (11)

where x_m_ is the mean of the 10 blank samples and SD is the standard deviation of the data. The LOD for Mg, Fe, Na, K and Ca in mg L^−1^ was 0.044, 0.020, 0.010, 0.005, and 0.079, respectively. On the other hand, the LOQ obtained for Mg, Fe, Na, K, and Ca in mg L^−1^ were 0.133, 0.060, 0.030, 0.015, and 0.237, respectively.

To evaluate the trueness of the procedure, a certified reference material (Tea Leaves INCT-TL-1) was used. To achieve this purpose, a series of six independent determinations were carried out, and the trueness of the method is presented as recovery values ranged between 95.5 and 107.3%. As it can be observed in Table 5, measured values are congruent to certified values provided by the certified reference material.

The precision was evaluated through the repeatability and intermediate precision. Regarding repeatability, a series of five determinations of the sample #W11 was held on the same day. The value is presented as the coefficient of variation (CV) for each metal, ranging between 7.3 and 9.7%. Finally, to determine the intermediate precision, a series of measurements was conducted on three different consecutive days, employing the sample #W11. The precision was presented as the CV average of the three consecutive days for each metal. These CVs corresponding to the intermediate precision ranged from 9.4 to 9.8%.

### 3.3. Essential Mineral Content

The determination of K, Fe, Mg, Ca, and Na in mushroom samples was carried out using the above-mentioned method, analyzing the samples in duplicate. The content of essential metals in each sample of mushroom is shown in Table 6. The results indicate that K was the predominant element in the mushroom samples, followed by Mg, while Fe and Na were found at the lowest levels.

*Magnesium*: For Mg determination, the average content in the mushroom samples was 1439 mg Kg^−1^, ranging from a minimum value of 939 mg Kg^−1^ to a maximum value of 2127 mg Kg^−1^. The highest Mg level was found in sample #W12: *P. ostreatus* (2127 ± 16.0 mg Kg^−1^) collected from Bouhachem (Tangier, Morocco). Conversely, the lowest Mg content was observed in sample #C5: *P. ostreatus* provided from Micotime (Spain) with 939 ± 35.2 mg Kg^−1^.

*Iron:* In the case of the determination of Fe, the values found in the samples varied between 35.7 mg Kg^−1^ to 608 mg Kg^−1^, with a mean value of 250 mg Kg^−1^. The mushroom with the highest Fe concentration was the sample *#W7: A. crocodilinus* (608 ± 4.51 mg Kg^−1^) from Fuente del Espino (Granada, Spain). In contrast, the lowest Fe content was detected in sample *#C1: A. bisporus* (35.7 ± 1.39 mg Kg^−1^) from Mercadona (Castilla la Mancha, Spain).

*Sodium:* On the other hand, the highest Na concentration was determined in sample *#C1: A. bisporus* (2167 ± 205 mg Kg^−1^) from Mercadona (Castilla la Mancha, Spain) whereas the lowest level was recorded in sample *#W9: A. silvícola* (28.8 ± 0.77 mg Kg^−1^) from Cortes de la Frontera (Malaga, Spain). The mean Na content across all samples was 455 mg Kg^−1^.

*Potassium:* According to the results, the K content in the mushrooms varied between 20,640 mg·Kg^−1^ to 55,086 mg Kg^−1^ with an average value of 38,562 mg Kg^−1^. The highest K concentration was measured in sample #W9: *A. silvicola* (55,086 ± 2027 mg Kg^−1^) from Cortes de la Frontera (Spain), while the lowest content was detected in sample #C5: *P. ostreatus* (20640 ± 649 mg Kg^−1^) obtained from Micotime (Cortes de la Frontera, Spain).

*Calcium*: Finally, regarding the results of Ca content, the mean value across the samples was 488 mg Kg^−1^. The highest level was found in sample *#C5: P. ostreatus* with a value of 1302 ± 59.0 mg Kg^−1^, collected from Micotime (Spain); meanwhile, the lowest was recorded in sample *#C3: P. ostreatus* (72.1 ± 1.94 mg Kg^−1^) from Mercadona (Castilla la Mancha, Spain).

According to the literature, the Mg content reported by other authors for the mushroom species analyzed in this study varied between 284 and 1990 mg Kg^−1^ [42,43,44,45,46,47]. Overall, these findings align with the Mg levels obtained in the present study, except for sample #W12, which slightly exceeds the upper limit on the reported range. Other authors have documented Fe levels in *Agaricus* spp., *Macrolepiota* spp. and *Pleurotus* spp. reporting values ranging from 26.0 to 644 mg Kg^−1^ [6,42,43,45,46,47,48]. Thus, it can be observed that the concentrations of this metal determined in this study are consistent with those found in the literature. On the other hand, the values of Na determined in the mushroom samples are in line with the concentrations documented in previous studies, which ranged from 16.0 to 3958 mg Kg^−1^ [6,42,43,44,45,46,47]. Regarding the content of K, some authors have analyzed the same species that were studied in this research, reporting values within a range of 136.0–45,860 mg Kg^−1^ [42,43,44,45,47]. Thus, it can be noted that the K content measured in this study exceeds the concentration reported by these authors. Finally, in relation to the Ca levels determined in this research, it is observed that they are in agreement with previous studies that establish a range of concentrations between 95.3 and 1940 mg Kg^−1^ [6,42,43,45,46,47].

Regarding the results obtained, the mushroom samples analyzed show a high content of several essential metals, which play a fundamental role in the correct development and functioning of the human body, especially Fe and K.

### 3.4. Recommended Daily Intake

The RDIs reference values for the different elements were provided by the Spanish Federation of Nutrition, Food and Dietetic Societies (FESNAD). For calculations, the RDI for adults aged from 20 to 59 years and for children aged from 10 to 13 years was used, as it is assumed that younger children do not typically consume a daily intake of 300 g of fresh mushrooms.

Considering the aforementioned, the estimated daily intake for each metal was calculated. Regarding the results, it can be seen that none of the mushroom samples satisfies the recommended daily intake for the metals determined on their own, except for Fe (Table 7). For this metal, samples #W11: *M. rhacodes* from Bouhachem (Tangier, Morocco) and #W12: *P. ostreatus* from Bouhachem (Tangier, Morocco) exceed the RDI values for men; samples #W6: *A. arvensis* from Bouhachem (Tangier, Morocco) and #W9: *M. mastoidea* from Bouhachem (Tangier, Morocco) exceed the RDI values for men and children; and sample #W7: *A. crocodilinus* from Fuente del Espino (Granada, Spain) exceeds the RDI for men, women, and children according to the FESNAD. Fe is the most abundant trace element in the body, found in many proteins, such as hemoglobin, as well as in various enzymes and cytochromes that play a role in redox reactions [49]. Thanks to its numerous oxidation states and its ability to form a wide range of coordination compounds, iron is widely distributed within the biological system, carrying out diverse functions [11]. As a result, an iron deficiency in the human body can lead to health issues, with anemia being the most prevalent. However, excessive levels of this metal can be considered toxic to human health, potentially causing gastrointestinal disorders, such as diarrhea or vomiting, and impairing the proper functioning of the liver, the central nervous system, or the cardiovascular system [11]. Comparing the results obtained in mushrooms with the concentration in other iron-rich foods such as eggs (34.4 mg Kg^−1^), chickpeas (55.0 mg Kg^−1^) or vegetables such as spinach (257 mg Kg^−1^), it can be observed that the concentration of iron found in mushrooms is higher [50,51,52]. Therefore, the results obtained show that some of the analyzed mushroom samples exceed the RDI levels for Fe. That fact highlights the high potential value for the development of food supplements based on the essential mineral content of these mushroom species.

In addition, a high percentage of the RDI of all the five elements determined is covered in the mushroom samples, except for Ca and Na, as shown in Table 8. Then, it should be noted that the daily intake of 300 g of fresh mushrooms covers an appropriate percentage of the recommended daily intake of each element, mainly for Fe, whose RDI is exceeded when certain species of mushrooms are ingested.

## 4. Conclusions

Due to mushrooms being important sources of nutrients and having a great potential as food supplements, the quantification of their mineral composition needs to be evaluated to permit an increase in their consumption. As far as we know, there are only a few reports focused on the essential mineral composition and the evaluation of the RDI. Thus, a successful optimization of a microwave-assisted digestion to determine the total content of Fe, Mg, K, Na, and Ca in mushroom samples has been carried out. The optimized method minimizes the nitric acid volume and the digestion time needed to destroy the organic matrix of mushrooms following the development of more environmentally friendly procedures. On the other hand, the analysis of the mushrooms studied shows that K is the macronutrient found at higher levels in the mushroom samples studied in this work. It should be highlighted that the content of Fe is higher compared to other foods known for their high iron concentration. Moreover, the intake of these mushroom samples covers a significant percentage of the RDI of most of the essential metals evaluated. This fact shows the elevated potential of mushrooms to be used as a source to manufacture food supplements achieving the mineral levels recommended by the health organizations.

## Figures and Tables

**Table 1:** List of all samples of mushrooms analyzed with their corresponding sample ID, species, specimen number (*n*), collection location and sampling year.

Sample ID	Specie	Location/Year of Collection
#C1	*Agaricus bisporus* (*n* = 13)	Castilla la Mancha (Spain) (Mercadona)/2021
#C2	*Agaricus bisporus* (*n* = 21)	Spain (LIDL)/2021
#C3	*Pleurotus ostreatus* (*n* = 23)	Castilla la Mancha (Spain) (Mercadona)/2021
#C4	*Pleurotus ostreatus* (*n* = 21)	Spain (DIA)/2021
#C5	*Pleurotus ostreatus* (*n* = 16)	Spain (Micotime)/2022
#W6	*Agaricus arvensis* (*n* = 24)	Bouhachem (Tangier, Morocco)/2019
#W7	*Agaricus crocodilinus* (*n* = 27)	Fuente del Espino (Granada, Spain)/2018
#W8	*Agaricus impudicus* (*n* = 15)	Sierra Alfaguara (Granada, Spain)/2020
#W9	*Agaricus silvicola* (*n* = 20)	Cortes de la Frontera (Malaga, Spain)/2018
#W10	*Macrolepiota mastoidea* (*n* = 19)	Bouhachem (Tangier, Morocco)/2019
#W11	*Macrolepiota rhacodes* (*n* = 11)	Bouhachem (Tangier, Morocco)/2019
#W12	*Pleurotus ostreatus* (*n* = 14)	Bouhachem (Tangier, Morocco)/2019

**Table 2:** Selected levels of each factor for the optimization method using the BBD.

Independent Variable	HNO_3_ (mL)	Time (min)	Temperature (°C)
Low level (−1)	5	10	100
Medium level (0)	7	15	150
High level (+1)	9	20	200

**Table 3:** Conditions of the different experiments for the optimization method using the BBD.

No. Experiment	HNO_3_ (mL)	Time (min)	Temperature (°C)
1	9	15	100
2	5	15	100
3	9	15	200
4	5	15	200
5	7	10	100
6	7	20	100
7	7	10	200
8	7	20	200
9	9	10	150
10	9	20	150
11	5	10	150
12	5	20	150
13	7	15	150
14	7	15	150
15	7	15	150

**Table 4:** Optimized FAAS instrumental parameters to determine Mg and Fe.

FAAS Parameters	Metal	
	Mg	Fe
Burner height (mm)	7.8	7.8
Fuel flow (L min^−1^)	0.9	1.1
Slit width (nm)	0.5	0.2
Wavelength (nm)	285.2	248.3

**Table 5:** Certified value (mg Kg^−1^) and measured value (mg Kg^−1^) of the certified reference material and the recovery percentage (%) of each metal.

Metal	Certified Value (mg Kg^−1^)	Measured Value (mg Kg^−1^)	Recovery Percentage (%)
Mg	2240 ± 170	2403 ± 208	107.3
Fe	432 ± 9.43	428 ± 9.14	99.1
Na	24.7 ± 3.2	23.6 ± 0.8	95.5
K	17,000 ± 1200	17,234 ± 1324	101.4
Ca	5820 ± 520	6171 ± 528	106.0

**Table 6:** Metal content in samples of mushroom analyzed (mg Kg^−1^ dry weight). Values are expressed as mean ± standard deviation (*n* = 2).

ID	Mg	Fe	Na	K	Ca
#C1	1563 ± 193	35.7 ± 1.39	2167 ± 205	52,136 ± 5627	395 ± 50.2
#C2	1219 ± 28.5	40.1 ± 1.17	894 ± 20.0	43,829 ± 858	299 ± 6.42
#C3	1470 ± 16.2	99.4 ± 2.00	110 ± 1.04	34,141 ± 95.9	72.1 ± 1.94
#C4	1542 ± 79.8	93.9 ± 3.63	68.5 ± 0.88	32,628 ± 1053	95.1 ± 4.32
#C5	939 ± 35.2	157 ± 6.91	734 ± 24.5	20,640 ± 649	1302 ± 59.0
#W6	1369 ± 12.7	580 ± 9.15	1052 ± 11.4	44,130 ± 115	831 ± 11.9
#W7	1325 ± 20.1	608 ± 4.51	187 ± 2.07	37,642 ± 626	1045 ± 6.77
#W8	1167 ± 5.02	302 ± 14.36	337 ± 1.53	36,749 ± 340	406 ± 9.08
#W9	1655 ± 100	106 ± 0.89	28.8 ± 0.77	55,086 ± 2027	110 ± 0.98
#W10	1339 ± 26.7	445 ± 20.9	83.2 ± 2.50	29,958 ± 373	400 ± 3.80
#W11	1306 ± 32.7	385 ± 13.7	70.3 ± 0.89	28,493 ± 523	195 ± 2.09
#W12	2127 ± 16.0	387 ± 18.1	502 ± 8.15	42,385 ± 250	818 ± 19.4

**Table 7:** Daily intake of metals in samples of mushrooms analyzed and recommended daily intake.

Metal	Estimated Daily Intake of Metals (mg/day)						Recommended Daily Intake (mg/day)		
	#C1	#C2	#C3	#C4	#C5	#W6	Children	Adults	
								Men	Women
**Mg**	39.7	35.0	49.7	39.2	46.8	46.8	280	350	300
**Fe**	1.10	1.23	3.00	2.84	4.72	17.4	12	9.5	18
**Na**	5.62	10.1	0.86	2.11	2.60	1.49	1500	1500	1500
**K**	1129	1102	1653	855	1238	1224	3100	3100	3100
**Ca**	31.3	12.2	3.31	5.85	13.2	12.7	1100	900	900
**Metal**	**Estimated Daily Intake of Metals (mg/day)**						**Recommended Daily** **Intake (mg/day)**		
	**#W7**	**#W8**	**#W9**	**#W10**	**#W11**	**#W12**	**Children**	**Adults**	
								**Men**	**Women**
**Mg**	36.6	44.1	41.1	63.8	40.2	46.9	280	350	300
**Fe**	18.2	9.20	3.20	13.3	11.5	11.5	12	9.5	18
**Na**	26.8	3.29	31.6	15.1	2.49	65.0	1500	1500	1500
**K**	1315	1024	1324	1272	899	1564	3100	3100	3100
**Ca**	8.98	2.16	24.9	24.5	12.0	11.9	1100	900	900

**Table 8:** Percentage contribution interval of RDI of each metal.

Metal	Percentage Contribution Interval of RDI (%)		
	Children	Men	Women
Mg	14–22	8–14	9–21
Fe	9–152	12–192	6–101
Na	0.1–4.3	0.1–4.3	0.1–4.3
K	28–53	28–53	28–53
Ca	0.2–3.6	0.2–4.3	0.2–4.3

## Data Availability

The original contributions presented in this study are included in the article. Further inquiries can be directed to the corresponding authors.
